# Yield, chemical composition, fermentation characteristics, in vitro ruminal variables, and degradability of ensiled amaranth (*Amaranthus hypochondriacus*) cultivars compared with corn (*Zea mays*) silage

**DOI:** 10.1093/tas/txaa180

**Published:** 2020-10-06

**Authors:** Hossein Shadi, Yousef Rouzbehan, Javad Rezaei, Hassan Fazaeli

**Affiliations:** 1 Department of Animal Science, Faculty of Agriculture, Tarbiat Modares University, Tehran, Iran; 2 Animal Science Research Institute of Iran, Agricultural Research, Education and Extension Organization (AREEO), Karaj, Iran

**Keywords:** amaranth silage, chemical composition, degradability, in vitro gas production, yield

## Abstract

Silages from four amaranth varieties (A5, A12, A14, and A28) were compared with corn silage (CS) in terms of their yield, chemical composition, phenolic compounds, oxalic acid and nitrate levels, silage fermentation characteristics, in vitro methane production, organic matter disappearance (OMD), microbial crude protein (MCP), ruminal ammonia (NH_3_-N), pH, volatile fatty acids, cellulolytic bacteria numbers, protozoa counts, and in situ dry matter (DM) and crude protein (CP) degradability were determined. Forages were harvested 93 d after planting, chopped, and ensiled in plastic buckets for 60 d. The study was based on a randomized complete block design, and data were analyzed using SAS, general linear model (GLM) procedure for normal distribution. Compared with CS, amaranth silages (AMS) had lower ash-free neutral detergent fiber nitrate, OMD (*P* < 0.001), phosphorus (*P* = 0.003), and metabolizable energy (ME) (*P* = 0.043) but higher (*P* < 0.001) CP, calcium, non-fiber carbohydrates (NFC), acid detergent lignin, ether extract, ash, total phenolics, pH, NH_3_-N concentration, MCP, digestible undegradable protein (DUP), and metabolizable protein (MP). Fresh, OM, OMD, ME (*P* < 0.001), and DM (*P* = 0.032) yields of AMS from different varieties were higher than CS, with the exception of A5. Overall, amaranth made good quality silage, with some variation, and A28 had the highest yield and nutritional value (CP, NFC, MCP, DUP, and MP). The yield, CP concentration, and nutritional value of A28 silage were higher than CS. Although these in vitro results are promising, they also need to be validated with future in vivo research.

## INTRODUCTION

Many amaranth varieties have been introduced into the Middle Eastern region, but their acclimation and the evaluation of their agronomic and nutritional values in the peculiar agroecologies of the region are unknown (Akin-Idowu et al., 2016). Amaranth has a lower water requirement than corn (Ofitserov, 2001) and can yield up to 85 t fresh weight/ha (Abbasi et al., 2012) with a higher crude protein (CP) (122 vs. 77 g/kg dry matter [DM]) and a lower acid detergent lignin (ADL) (35 vs. 45 g/kg DM; Rezaei et al., 2015). The CP in most forages is predominantly rumen degradable protein (RDP). However, Lotfi et al. (2018) showed that amaranth silages (AMS) had higher CP levels than corn silage (CS) and particularly more digestible undegradable protein (DUP). The DUP is available in the lower gut and is more efficiently used in post-ruminal digestion (Van Soest, 1994). Increasing DUP in heifer diets improved feed efficiency and live weight gain (Tomlinson et al., 1997) and dairy cow milk yield (Vagnoni and Broderick, 1997). Low protein solubility feeds with high levels of RUP were reported to result in lower methane production compared with high protein solubility feeds, that is, fermentation of rumen protein resulting in methane production (Preston et al., 2013; Ho Quang Do et al., 2013).

Genotypes of *Amaranthus* are characterized by fast growth after germination (Pisarikova et al., 2006), early maturity, water requirement (Kauffman and Weber, 1990), and high nutritive value of AMS as a ruminant feed (Rezaei et al., 2009). This has led to its use as a substitute for corn, especially in arid regions (Rezaei et al., 2015). Replacing CS with AMS had a similar effect on Holstein dairy cow performance (Rezaei et al., 2015) and improved the growth rate of lambs (Rezaei et al., 2014). Hence, testing different varieties of AMS to select the best of them is desirable. Therefore, in this study, four new varieties of amaranth considered suitable for silage production in semi-arid zones were chosen and their silage characteristics, including yield, chemical composition, antinutrient levels, silage fermentation characteristics, in vitro digestibility, and in situ DM and CP degradability, were compared with those of CS.

## MATERIALS AND METHODS

The *Guide for the Care and Use of Agricultural Animals in Research and Teaching* (FASS, 2010) was followed for housing, feeding, transport, proper and humane care and use of animals, veterinary care, occupational health and safety, program management, and procedures. The Committee of Animal Science of Tarbiat Modares University (Iran) approved the experimental protocols.

### Forage and Silage Preparation and Sampling Method

Corn (*Zea mays*, var. hybrid SC 704; Seed and Plant Research Improvement Institute, Karaj, Iran) and four amaranth varieties (A5, A12, A14, and A28) provided by S.-E Jacobsen (Quinoa Quality ApS, Teglværksvej 10, DK-4420 Regstrup, CVR 40610588). According to the agronomic performance of these varieties, they were selected by S.-E. Jacobsen, who is a member of the European project entitled PROTIEN2FOOD and working on amaranth and Quinoa plants, which ended in February 2020. The cultivated species of amaranth (*Amaranthus* sp.) originated from Latin America and belong to the species such as *Amaranthus hypochondriacus*, *A. caudatus*, and *A. cruentus*. The corn and four amaranth varieties were grown near the city of Nishapur (Khorasan-e Razavi Province, Iran) at 1,250 m above sea level, with an average yearly (2017) rainfall of 250 mm and an average temperature of 20 °C in a soil characterized as soft loam. A randomized complete block design with four replicates (four plots of 25 m^2^ each per treatment) was used in the field study. The forages were sown on April 30 (2017). Amaranth cultivation was initially conducted in special culture trays (industry standard) with a coconut coir bed and then planted out when the plants reached 10 cm height (industry standard). The corn was sown with a four-row precision drill (Tarashkadeh Co., Karaj, Iran). All plants were spaced in 50-cm rows. There was daily irrigation of amaranth plants growing in coco coir beds and every 2 d during the vegetative period in the field plots in the form of droplets with special drip irrigation strips (20 cm hole spacing, bar type). The mean total water volumes applied to the amaranths and corn were 250 and 350 mm (i.e., 2,500 and 3,500 m^3^/ha), respectively. Ninety-three days after planting, the forages were harvested leaving a 10-cm stubble, and the corn was harvested at the milk stage of its kernels (growth stage BBCH-75; Lancashire et al., 1991). The forages were chopped into approximately 2-cm length cut. Homogeneous mixtures of each of the five treatments were packed tightly into 20-liters plastic barrels, and excess air was removed before the barrels were vacuumed to remove excess air before sealing to maintain the anaerobic environment. The barrels were ensiled for 60 d (one for each plot), and individual samples weighing 3 kg were taken before (i.e., fresh samples were taken per silo) and after ensiling for later analyses.

For measuring silage pH, 50 g of fresh silage was blended with 125 mL of distilled water and allowed to stand at room temperature for 1 h (Faithfull, 2002). After decanting the silage extract into a small beaker, the pH was measured using a digital pH meter (Sartorius PT-10; Germany). Two milliliters of juice from the silages were pipetted into centrifuge tubes containing 0.2 mL of acid (25% meta-phosphoric acid and 2-ethyl butyric acid 2 g/L as the internal standard), then centrifuged at 10,000 × *g* for 10 min at 4 °C (Galyean, 1997). Volatile fatty acids in the supernatant were quantified using gas chromatography (UNICAM 4600; SB Analytical, Cambridge, UK) with a flame ionization detector (FID; 250 °C), split-injection port (1.0 μL injection), capillary column (Agilent J & W HP-FFAP, 10 m by 0.535 mm by 1.00 μm, 19095F-121; Agilent, CA), and helium as the carrier gas (column head pressure of 10 psi). To determine NH_3_-N, an extract was obtained by squeezing the silage material, filtered using Whatman 54 filter paper, then a 9 mL of aliquot was taken, mixed with 1 mL of 7.2 N H_2_SO_4_, and stored at −20 °C. After thawing, the silage extracts were analyzed for NH_3_-N using a phenol-hypochlorite assay (Galyean, 1997).

### Chemical Analyses

Weighed samples were dried at 60 °C for 48 h period to determine DM concentration, ground to 1-mm sieve (Wiley mill; Thomas Scientific, Gloucester, NJ) and analyzed for organic matter (OM) (method 924.05), CP (method 984.13), ether extract (EE; method 954.02), ash-free acid detergent fiber (ADFom), and ADL (method 973.18) by AOAC (1998) procedures. Ash-free neutral detergent fiber (NDFom) was determined by Mertens (2002) method. The determination of water-soluble carbohydrates (WSC) concentration was carried out using the anthrone reaction assay, and absorbance of the extract was measured by a spectrophotometer (MAFF, 1986). The colorimetry was used to measure nitrate (Singh, 1988). The levels of oxalic acid were measured using a spectrophotometer after extracting the total oxalate from 1 g of ground sample with 50 mL 2 M HCl at 80 °C for 15 min (Savage et al., 2000). The Folin–Ciocalteau method (Makkar, 2000) was used to measure total (TP) and non-tannin phenolics (NTP). The difference between TP and NTP gives the amount of total tannins (TT) with tannic acid (Merck GmbH, Darmstadt, Germany) used as the standard.

### In Vitro Gas Production and Related Variables

In vitro gas production (GP) was carried out for 24 h (Menke et al., 1979) to assess treatment GP and fermentation variables (pH, NH_3_-N, lactate, volatile fatty acids, cellulolytic bacteria and protozoa numbers, and methane). A probe used for ruminal fluid collection from the rumen of three fistulated adult Shall sheep before the morning feeding. The sheep’s diet contained 10%, 15%, 40%, 14%, 15%, 5%, and 1% of AMS, CS, alfalfa hay, wheat bran, rolled barley, soybean meal, and a vitamin–mineral mix, respectively (on a DM basis). Sheep feeding times were 0700 and 1900 h, and they had free access to water. After filtering through cheesecloth (four layers), the rumen fluid was mixed with an anaerobic mineral buffer (1:2, v/v) in CO_2_-flushed thermos flasks warmed to 39 °C and stirred under CO_2_ until use (Menke et al., 1979). Each silage sample of 200 mg was incubated for 24 hours at 39 °C in a prewarmed 100-mL glass syringe containing 30 mL of rumen fluid and buffer (1:2, v/v). One hundred and sixty-six syringes (five treatments × four blocks [replicates] × two individual samples per block [replicate] × two syringes per sample × two runs, with three blanks in each run) were used in total. After 24 h, the volume of gas (GP) was measured, and the disappearance of OM (OMD) and metabolizable energy (ME) were estimated as follows (Menke et al., 1979): 


$$\begin{array}{l} \begin{array}{ll} & OMD\;\left(g/kg\right)=148.8+\left(8.893\times GP_{24}\right)+\left(0.448\times CP\right)\\ & \;+\left(0.651\times XA\right)\\ & ME\left(MJ/kg\;DM\right)=2.20+\left(0.1357\times GP_{24}\right)\\ & \;+\left(0.0057\times CP\right)\\ & \;+\left(0.00002859\times CP^{2}\right) \end{array} \end{array}$$

In the above equations, OMD is the disappearance of OM, GP_24_ is net gas produced over 24 h (mL/200 mg DM), CP is crude protein in g/kg DM, XA is ash in g/kg DM, and ME is the metabolizable energy.

Truly degraded substrate (TDS), partitioning factor after 24 h (PF_24_), and microbial CP (MCP) of the treatments were measured by taking the contents of eight syringes per treatment from each run (four blocks × two individual samples × one syringe per sample), removing soluble products using a neutral detergent solution (Van Soest et al., 1991) and then weighing the undissolved feedstuffs in crucibles, after washing and drying at 60 °C for 48 h. Loss in weight after drying was the measure of TDS (mg/g DM; Blümmel et al., 1997). The PF_24_ (to estimate the efficiency of fermentation) was derived from the equation: $PF_{24}\left(mg/mL\right)\;=\;TDS\;\left(mg\right)/GP_{24}\left(mL\right)$, where PF_24_ is the partitioning factor after 24 h (mg substrate truly degraded in vitro/mL gas) and GP_24_ is gas produced after 24 h (Blümmel et al., 1997). The MCP (mg/g DM) was derived from the equation: $TDS\;\left(mg\right)\;-\;(mL\;GP_{24}\times \;2.2\;mg/mL)$, the 2.2 mg/mL being a stoichiometric factor expressing mg of carbon, hydrogen, and oxygen required for the short-chain fatty acids–gas complex production needed for 1 mL of GP (Blümmel et al., 1997).

Eight syringes per treatment per run (four blocks × two samples × one syringe per sample) were used to determine in vitro pH, volatile fatty acids (VFA), NH_3_-N, cellulolytic bacteria, and protozoa numbers. After 24 h, the pH of the syringe contents was measured with a digital pH meter (Sartorius PT-10, Germany). A strained sample of 2.5 mL was mixed with 0.5 mL of 0.2 N HCl and analyzed for NH_3_-N using the phenol-hypochlorite assay (Galyean, 1997). For analysis of VFAs, 2 mL of supernatants was preserved, at –20 °C, with 0.5 mL of an acid solution containing 20% orthophosphoric acid and 20 mM 2-ethylbutyric acid. Total VFAs were measured by gas–liquid chromatography using ethyl-butyric acid as the internal standard. Counting total and subfamily numbers of protozoa from syringe contents was done using the method of Dehority (2003); 5 mL of syringe contents, which was strained through three layers of cheesecloth into a CO_2_-filled sterilized bottle (39 °C), was used to enumerate the cellulolytic bacteria population. The anaerobic techniques of Hungate (1966) as modified by Bryant (1972) were used to prepare anaerobic culture media. Hungate tubes with anaerobic media and Whatman number 1 filter paper, as the carbohydrate source, were made. Strained rumen fluid was serially diluted and added to the tubes that were incubated at 39 °C for 21 d.

Methane produced in the syringes was determined using the method of Anele et al. (2011). The total GP_24_ from each syringe was measured, then 4 mL of 10 M NaOH was added to each syringe to absorb CO_2_, and then the remaining gas volume was recorded as CH_4_.

The GP kinetics of the treatments were evaluated using a 96-h in vitro GP. Gas volumes were measured at 2, 4, 6, 8, 10, 12, 16, 24, 48, 72, and 96 h. Kinetic parameters were estimated using the exponential model described by Blümmel et al. (2003), $y=B(1\;\;e^{-ct})$, where *y* is the volume of gas at time *t*, *B* is the asymptotic value of GP (mL/200 mg DM), and *c* is the first-order fractional constant rate of GP (/h).

### In Situ Degradability of DM and CP

The degradability of DM and CP of the samples was calculated according to the nylon bag method, pre-weighed bags were placed in the rumens of four fistulated male Shal sheep of 62 ± 2.1 kg live weight (AFRC, 1992). The sheep’s diet contained 10%, 15%, 40%, 14%, 15%, 5%, and 1% of AMS, CS, alfalfa hay, wheat bran, rolled barley, soybean meal, and a vitamin–mineral mix, respectively (on a DM basis), fed twice daily at 0700 and 1900 h as a total mixed ration, and with 24 h access to water. The bag size was 21 × 10 cm with a pore size of 45 µm (Bucksburn, Aberdeen, UK). Dried silage samples were ground to 4-mm sieve in a Cyclotec TM 1093 Sample Mill (Foss Companies, Hillerød, Denmark). Samples of 5-g DM were placed into bags and incubated for 2, 4, 8, 12, 24, 48, 72, and 96 h. One bag per sample per sheep for each time was used. Following incubation time, bags were removed by hand and rinsed under tap water, washed in a washing machine (Hoover OPHS 612; London, UK) using cold water for 1 h, and then dried in a 55 °C oven for 48 h. Time zero degradability was measured by cold water machine washing three extra bags per sample for 1 h. Bags and contents were weighed to estimate degraded DM. The percentage of ruminal degradability (*Y*) at time (*t*) was obtained from an exponential curve of the type $Y=a+b\;(1\;\;{e^{}}^{ct})$, which was fitted to the experimental data by iterative regression analysis (Ørskov and McDonald, 1979). In this equation, e is the base of natural logarithms, the constant a represents the soluble and very rapidly degradable fraction, and b represents the insoluble but potentially degradable fraction, which degrades at a constant fractional rate (*c*) per unit time. Effective DM degradability (ED) in the silages was then estimated (Ørskov and McDonald, 1979) as:

ED = (a+b×c)/(c+k) , in which ED is the effective DM degradability; constant *a* is the soluble and very rapidly degradable fraction; *b* is the insoluble but potentially degradable fraction, which degrades at a constant fractional rate (*c*) per unit time; and k is the fractional outflow rate of small particles from the rumen. An assumed value of *k* = 2%/h was used, which is an average value for sheep given a mixed diet at a very low level of feeding, equivalent to approximate maintenance (Alderman et al., 1993). CP from bag residues was measured using the method No. 988.05 of AOAC (1998). The ruminal degradability percentage (Y) of DM and CP at time (*t*) was taken from an exponential curve where $Y\;=\;A\;+\;B\;(1-{e^{-}}^{Ct})$, which fitted the experimental data using iterative regression analysis (Ørskov and McDonald, 1979). The ED of DM and CP was then estimated (Ørskov and McDonald, 1979) as ED (%) = (A + B × C)/ (C + k). In these equations, “e” is the base of natural logarithms, “A” is the soluble and very rapidly degradable fraction, and “B” represents the insoluble but potentially degradable CP fraction, which degrades at a constant fractional rate (c) per unit time (t), ED is the effective degradability, and “k” refers to the fractional outflow rate from the rumen. An accepted value for “k” was 0.02 fraction/h (Alderman et al., 1993). Effective RDP (ERDP), DUP, and metabolizable protein (MP) levels were calculated from equations recommended by AFRC (1992).

### Statistical Analysis

Data on the yield, chemical composition, and silage fermentation characteristics were analyzed using the GLM procedure of SAS (SAS Inst. Inc., Cary, NC) in a randomized complete block design with the fixed effect of treatment. The model was $Y_{ijk}=\mu +\;T_{i}+\;B_{j}+\;e_{ij}+\;e_{ijk}$, where Y_ijk_ is observation, μ is general mean, T_i_ is treatment effect, B_j_ is block effect, e_ij_ is experimental error, and e_ijk_ is sampling error.

A split-plot in a randomized complete block design (including the effects of treatment, block [replicate], sample per block, and run) was used for analyzing the in vitro GP data, where treatment was considered as the main plot and run as the subplot. The analysis was performed based on the model $Y_{ijkl}=\mu +\;T_{i}+\;B_{j}+\;e_{ij}+\;R_{k}+\;{\left(TR\right)}_{ik}+\;e_{ijk}+\;e_{ijkl}$ In this model, $Y_{ijkl}$, μ, $T_{i}$, $B_{j}$, $e_{ij}$, $R_{k}$, ${\left(TR\right)}_{ik}$, $e_{ijk}$, and $e_{ijkl}$ are observation, general mean, treatment effect, block effect, treatment × block (main plot error), run effect, treatment × run, treatment × block × run (split-plot error), and treatment × block × run × sample (sampling error), respectively.

Prior to ANOVA, residual normality for data was tested using Proc UNIVARIATE. Multiple comparisons among the means were performed with the Tukey’s multiple range test. Statistical significance was defined by *P*-values ≤ 0.05.

## RESULTS

### Yield and Chemical Composition

The yield of the forages before and after ensiling is presented in Table 1. When comparing yields per hectare of pre-ensiled corn and amaranth varieties, it was found that A28 had the highest fresh weight (*P* < 0.001), DM (*P* = 0.032), OMD, CP, and MP (*P* < 0.001).

**Table 1.** Yields of the fresh forages and silages of four amaranth varieties (A5, A12, A14, and A28) and corn

		Amaranth					
Item	Corn	A5	A12	A14	A28	SEM	*P*-value
Yield, t/ha							
Fresh forage	35.0^d^	19.0^e^	44.2^c^	51.0^b^	56.0^a^	1.20	<0.001
Ensiled forage							
DM	9.10^c^	4.91^d^	11.8^ab^	12.9^ab^	14.1^a^	0.47	0.032
OM	8.40^b^	4.01^c^	9.58^ab^	10.3^a^	11.0^a^	0.52	<0.001
OMD	5.68^a^	2.23^b^	5.22^a^	6.02^a^	6.13^a^	0.50	<0.001
CP^*1*^	0.683^d^	0.351^e^	1.92^b^	1.52^c^	2.57^a^	0.03	<0.001
MP	0.410^d^	0.225^e^	1.23^b^	0.984^c^	1.59^a^	0.008	<0.001

^*1*^CP estimated from total nitrogen content × 6.25.

Means in the same row with different superscripts (a–d) letters are significantly different (*P* < 0.05).

Freshly harvested corn had higher (*P* < 0.001) NDFom, ADFom, WSC, and phosphorus concentrations compared with the amaranth forages (Table 2). CS had higher NDFom, ADFom, non-fiber carbohydrates (NFC) (*P* < 0.001), and phosphorus (*P* = 0.003) levels compared with AMS. The cultivar A28 had the highest CP level (*P* < 0.001) compared with all other forages and silages. Among the amaranth varieties, A12 had the highest (*P* < 0.001) NDFom level, and A5 had the lowest (*P* < 0.001) ADFom, ADL, and ash concentrations but higher concentration (*P* < 0.001) of EE compared with the other amaranth forages and silages. Moreover, the WSC concentration of the fresh forage (*P* < 0.001) and silage (*P* = 0.028) from variety A5 was higher than the other amaranth varieties.

**Table 2.** Chemical composition (g/kg DM) of the fresh forages and silages of four amaranth varieties (A5, A12, A14, and A28) and corn

	Fresh forages							Ensiled						
	Amaranth							Amaranth						
Item	Corn	A5	A12	A14	A28	SEM	*P*-value	Corn	A5	A12	A14	A28	SEM	*P*-value
DM^*a*^	252	251	258	246	247	4.8	0.12	260	258	267	253	251	7.0	0.13
CP^*b*^	80^d^	83^d^	176^b^	129^c^	192^a^	1.9	<0.001	75.0^d^	81.4^d^	163^b^	118^c^	182^a^	1.8	<0.001
NDFom	515^a^	402^b^	413^b^	373^c^	376^c^	4.0	<0.001	490^a^	391^b^	405^b^	361^c^	367^c^	4.2	<0.001
ADFom	318^a^	246^d^	273^c^	249^d^	289^b^	3.3	<0.001	292^a^	235^d^	265^c^	239^d^	281^b^	2.3	<0.001
ADL	51.0^c^	47.3^d^	61.3^b^	59.3^b^	64.6^a^	1.0	<0.001	48.7^c^	53.0^b^	59.3^ab^	56.0^b^	62.0^a^	1.3	<0.001
WSC	111^a^	75.5^b^	59.6^d^	63.3^c^	53.8^d^	0.73	<0.001	15.2^ab^	15.1^b^	11.0^c^	15.0^b^	14.5^b^	0.87	0.028
EE	32.5^c^	44^a^	40^b^	41.5^b^	42^b^	0.93	<0.001	42.5^c^	90.3^a^	81.6^b^	77^b^	77^b^	1.1	<0.001
NFC	310^a^	321^a^	209^c^	269^b^	200^c^	2.89	<0.001	317^a^	265^b^	163^d^	241^c^	159^d^	1.8	<0.001
Ash	63^e^	150^c^	163^b^	188^a^	190^a^	1.1	<0.001	76.0^e^	182^d^	188^c^	203^b^	215^a^	0.90	<0.001
Calcium	4.5^b^	12.1^a^	11.6^a^	12.4^a^	12.0^a^	0.49	<0.001	4.6^b^	12.1^a^	11.8^a^	12.7^a^	12.0^a^	0.48	<0.001
Phosphorus	7.1^a^	4.0^b^	3.8^b^	3.7^b^	3.6^b^	0.42	<0.001	7.1^a^	4.0^b^	3.8^b^	3.8^b^	3.6^b^	0.38	0.003
Magnesium	3.5	3.5	3.7	3.9	3.6	0.28	0.11	3.7	3.6	3.8	3.8	3.8	0.19	0.19

^*a*^DM (g/kg as fed).

^*b*^CP estimated from total nitrogen content × 6.25; ADL, lignin measured after solubilization of cellulose with 72% sulfuric acid.

Means in the same row with different superscripts (a–d) letters are significantly different (*P* < 0.05).

Amaranth forage varieties had higher (*P* < 0.001) nitrate, TP, and TT concentrations than corn (Table 3). Among the silages, CS had the highest nitrate concentration, A12 had the highest oxalate and TP concentration, and A28 had the highest TT concentration (*P* < 0.001).

**Table 3.** Antinutritional factors levels (g/kg DM) of the fresh forages and silages of four amaranth varieties (A5, A12, A14, and A28) and corn

	Fresh forages							Ensiled						
	Amaranth							Amaranth						
Item	Corn	A5	A12	A14	A28	SEM	*P*-value	Corn	A5	A12	A14	A28	SEM	*P*-value
Nitrate	2.1^e^	2.5^c^	2.3^d^	3.5^a^	3.0^b^	0.03	<0.001	0.69^a^	0.50^b^	0.35^c^	0.51^b^	0.49^b^	0.01	<0.001
Oxalate	7.3^b^	8.6^b^	13.0^a^	4.7^c^	8.8^b^	0.6	<0.001	7.2^b^	8.4^b^	12.0^a^	4.5^c^	8.9^b^	0.60	<0.001
TP	3.5^e^	8.6^d^	9.8^b^	9.0^c^	10.0^a^	0.02	<0.001	2.0^e^	4.1^d^	7.0^a^	6.2^b^	5.7^c^	0.02	<0.001
TT	2.0^e^	5.0^d^	5.9^b^	5.3^c^	6.4^a^	0.04	<0.001	1.2^e^	1.7^d^	4.0^b^	2.5^c^	4.9^a^	0.03	<0.001

Means in the same row with different superscripts (a–d) letters are significantly different (*P* < 0.05).

### Silage Fermentation Characteristics

Ammonia-N concentration and pH were higher (*P* < 0.001) in AMS, compared with CS, their values ranging from 54.5 to 60 g/kg of total N and 4 to 4.6, respectively (Table 4). Among AMS, A5 had the lowest (*P* < 0.001) pH and NH_3_-N concentration.

**Table 4.** Fermentative characteristics of the corn and AMS varieties (A5, A12, A14, and A28)

	Amaranth						
Item	Corn	A5	A12	A14	A28	SEM	*P*-value
pH	3.8^e^	4.0^d^	4.2^c^	4.4^b^	4.6^a^	0.057	<0.001
NH_3_-N, g/kg total N	39.5^c^	54.5^b^	58.0^a^	59.5^a^	60.0^a^	0.8	<0.001
Lactic, g/kg DM	65.2	63.0	61.0	59.1	58.5	4.7	0.64
Acetic, g/kg DM	19.0	17.8	16.7	16.4	16.0	1.01	0.91
Propionic, g/kg DM	3.2	3.2	3.9	4.2	4.4	0.6	0.70
Butyric, g/kg DM	0.21	0.25	0.40	0.46	0.52	0.04	0.73

Means in the same row with different superscripts (a–c) letters are significantly different (*P* < 0.05).

### In Vitro GP and Fermentation Variables

The in vitro ruminal pH did not differ (*P* = 0.93) among ensiled forages, but the level of NH_3_-N was lower in CS (*P* < 0.001), compared with AMS (Table 5). The incubated CS had higher in vitro total VFA concentration (*P* = 0.005) and cellulolytic bacteria numbers (*P* = 0.003) compared with the other silages, with no difference in total protozoa. AMS had higher (*P* = 0.008) Entodiniinae protozoa count than CS, but CS and A5 had higher (*P* = 0.009) Isotrichidae protozoa count than other silages. CS had higher GP_24_ OMD, TDS (*P* < 0.001), ME (*P* = 0.043), and in vitro methane production (*P* < 0.001) than AMS (Table 6). AMS had higher (*P* < 0.001) in vitro ruminal MCP compared with CS. However, there was no difference (*P* = 0.060) among AMS in PF_24_. The in vitro incubation of A12 and A28 resulted in lower methane production (*P* < 0.001) than the other AMS.

**Table 5.** In vitro ruminal fermentation characteristics, ruminal protozoa numbers (× 10^5^/mL digesta), and cellulolytic bacteria numbers (log_10_/g digesta) of the AMS varieties (A5, A12, A14, and A28) and corn

		Amaranth					
Item	Corn	A5	A12	A14	A28	SEM	*P*-value
pH	6.72	6.72	6.76	6.79	6.80	0.58	0.93
NH_3_-N, mg/dL	11.7^d^	14.8^c^	15.5^c^	17.3^a^	16.3^b^	0.23	<0.001
Total VFA, mmol/L	63.0^a^	57.5^b^	53.9^b^	57.0^b^	53.5^b^	1.4	0.005
VFA, mol/100 mol							
Acetate	72.5	68.6	70.0	70.6	69.6	5.7	0.59
Propionate	20.2	21.5	20.8	20.5	21.3	2.5	0.88
Butyrate	5.6	7.7	7.1	6.1	6.8	0.9	0.38
Isovalerate	1.7	2.2	2.5	2.8	2.3	0.44	0.97
Total protozoa	6.68	6.78	6.66	6.78	6.60	0.08	0.59
Isotrichidae	1.93^a^	1.92^a^	1.60^bc^	1.80^ab^	1.51^c^	0.07	0.009
Entodiniinae	2.81^e^	3.03^d^	3.11^bc^	3.08^cd^	3.25^a^	0.029	0.008
Diplodiniinae	1.12	1.01	1.15	1.08	1.02	0.06	0.57
Ophrioscolecinae	0.823	0.823	0.798	0.822	0.823	0.024	0.97
Cellulolytic bacteria	8.8^a^	8.2^b^	8.3^b^	8.4^b^	8.2^b^	0.086	0.003

Means in the same row with different superscripts (a–d) letters are significantly different (*P* < 0.05).

**Table 6.** In vitro ruminal GP and estimated parameters, TDS, and MCP of the AMS varieties (A5, A12, A14, and A28) and corn

	Amaranth						
Item	Corn	A5	A12	A14	A28	SEM	*P*-value
24-h incubation							
GP_24_, mL/200 mg DM	50^a^	29^b^	22.5^c^	28^b^	21^c^	0.88	<0.001
OMD, g/kg	676^a^	557^c^	545^c^	584^b^	557^c^	5.7	<0.001
ME, MJ/kg DM	9.57^a^	6.69^b^	6.95^b^	7.08^b^	6.93^b^	1.18	0.043
TDS, g/kg DM	658^a^	625^c^	635^b^	632^b^	614^d^	2.6	<0.001
PF_24_, mg TDS/mL GP_24_	2.63^b^	4.31^a^	5.64^a^	4.80^a^	5.84^a^	0.88	0.060
MCP, g/kg DM	108^e^	306^d^	388^a^	364^c^	383^b^	1.95	<0.001
In vitro methane, mL/g DM	29.5^a^	25.6^b^	21.5^c^	25.0^b^	20.0^c^	0.68	<0.001
96-h incubation^*a*^							
B, mL/200 mg DM	57.3^a^	50.0^b^	40.0^d^	42.1^c^	50.0^b^	0.51	<0.001
C, /h	0.030^b^	0.034^b^	0.034^b^	0.070^a^	0.035^b^	0.002	<0.001

^*a*^B, the asymptotic value of gas production; C, constant rate of gas production.

Means in the same row with different superscripts (a–d) letters are significantly different (*P* < 0.05).

### In Situ Degradability of DM and CP

There were differences in CP and DM degradation in CS and amaranth varieties (Table 7). Among all silages, A5 had the highest (*P* < 0.001) ED of DM. The ED of CP for A28 was the highest among AMS, but they were all lower (*P* < 0.001) than that of CS. The cultivar A28 had higher (*P* < 0.001) ERDP and MP in comparison with the other silages, and the DUP values of A12 and A28 were higher (*P* < 0.001) than the other AMS.

**Table 7.** In situ degradability of DM and CP of the silages (four amaranth varieties and corn)

		Amaranth					
Item	Corn	A5	A12	A14	A28	SEM	*P*-value
DM degradation^*1*^							
A, g/kg DM	420^a^	425^a^	373^b^	436^a^	378^b^	8.6	<0.001
B, g/kg DM	340^c^	408^b^	354^b^	353^b^	535^a^	8.5	<0.001
C, /h	0.038^c^	0.062^a^	0.380^c^	0.040^b^	0.014^d^	0.008	<0.001
ED, g/kg DM	602^b^	632^a^	573^c^	583^bc^	509^d^	6.53	<0.001
CP degradation							
A, g/kg CP	460^a^	361^d^	334^e^	387^c^	426^b^	6.9	<0.001
B, g/kg CP	324^d^	347^c^	372^b^	330^cd^	454^a^	6.9	<0.001
C, /h	0.041^a^	0.030^b^	0.029^b^	0.017^c^	0.019^c^	0.006	<0.001
ADIN	1.10^c^	1.16^c^	2.42^a^	1.75^b^	2.66^a^	0.1	<0.001
ED, g/kg CP	605^a^	492^c^	480^c^	471^c^	550^b^	6.8	<0.001
ERDP, g/kg DM	38.5^d^	30.2^e^	67.1^b^	46.3^c^	85.1^a^	1.01	<0.001
DUP, g/kg DM	20.0^d^	26.5^c^	62.7^a^	46.2^b^	59^a^	1.21	<0.001
MP, g/kg DM	45.1^d^	46.0^d^	105^b^	76.3^c^	113^a^	1.27	<0.001

^*1*^A, soluble and very rapidly degradable fraction; B, insoluble but potentially fermentable fraction; C, fractional degradation rate of B; ED calculated for an outflow rate of 0.02/h.

Means in the same row with different superscripts (a–d) letters are significantly different (*P* < 0.05).

## DISCUSSION

### Yield and Chemical Composition

Among all the forages, the yields of A12, A18, and A28 were relatively good (11.8 to 14.1 t/ha on a DM basis; Table 1). Yields of up to 86.4 t/ha as fed and 13.2 t/ha of DM have been recorded for amaranth forage, respectively (Mehrani et al., 2012), with differences in yield being attributed to differences in variety, soil, climate, season, and N fertilizer usage (Klemencic and Kramberger, 2006).

Differences in the nutritional values of the amaranth varieties compared with corn are reflected in their chemical composition. The amaranth forage DM (>25% of fresh weight) in this study was more than that observed by Rezaei et al. (2014), but was within the range reported by Abbasi et al. (2018). The lowest level (8% of DM) of CP needed to provide the necessary level of NH_3_-N to support optimum rumen microbiota growth (Norton, 1998) was found in all AMS suggesting that they have potential as a CP source in ruminant rations.

Compared with CS, AMS had lower levels of NDFom and ADFom showing their potential for ruminant fodder (Rezaei et al., 2014), as these compounds (plant cell wall derivatives) in high concentrations limit feed intake and energy availability (Jung and Allen, 1995). Seguin et al. (2013) showed that the cell wall contents of AMS fairly similar to our findings (361 to 405 g/kg DM), whereas Olorunnisomo and Ayodele (2009) and Rezaei et al. (2015) reported higher NDFom levels. The ADL concentrations in these AMS varieties (46 to 65 g/kg DM) were higher than those reported for AMS from other varieties (Seguin et al., 2013; Rezaei et al., 2015). Differences in ADL depend on the growth stage at harvest and the cultivar (Abbasi et al., 2012). Fermentation requires an adequate WSC concentration for conversion into lactic acid to lower pH for good quality silage (Kaiser et al., 2004). Forages with levels of 50 to 80 g WSC/kg DM should develop low pH sufficient for a stable silage (McDonald et al., 1991). In the current study, all pre-ensiled forages had WSC concentrations above 50 g/kg DM, and good stable silages were achieved. Low levels of residual WSC in the silages indicate increased bacterial utilization leading to higher lactic acid production. After ensiling, reductions in WSC, protein, and NDF led to a proportional increase in silage EE levels. Similar to the present study, Abbasi et al. (2012) and Seguin et al. (2013) reported high ash concentration in amaranth. The ash content in AMS (182 to 215 g/kg DM) was genetically higher than that of CS, which was comparable to the results reported by the others (Olorunnisomo and Ayodele, 2009; Karimi-Rahjerdi et al., 2015). Compared with CS, AMS had higher Ca and lower phosphate concentrations. These Ca levels were lower than in other studies (Rezaei et al., 2014), and the Ca:P ratio in AMS, which ranged from 2.5:1 to 3.3:1, was higher than the recommended 2:1 ratio. However, a Ca:P ratio of 2:1 or greater is recommended to prevent urinary calculi in sheep (NRC, 1985). The amaranth varieties had higher Mg concentrations compared with Plainsman and D136 varieties, but these differences may be caused by other changes in factors that influence plant growth (Seguin et al., 2013).

The amaranth forage had higher levels of nitrate than corn but nitrate concentration decreased over time following a decline in CP (i.e., from 253 to 160 g/kg DM at 40 and 60 d after planting, respectively), similar to that reported by Sleugh et al. (2001). Ensiled amaranth had lower nitrate levels than the fresh forage, because of the action of microorganisms reducing nitrate to NH_3_-N via nitrite in the ensiling process (McDonald et al., 1991). The nitrate content of AMS, at 0.3 to 0.69 g/kg DM, was lower than the toxic level for ruminants (i.e., > 6 g/kg DM; Radostits et al., 2007).

High levels of oxalate ingestion can cause hypocalcemia and kidney failure, and high doses can prove fatal (Knight and Walter, 2003). In this study, the ensiling of amaranth had no effect on oxalic acid concentration with oxalate in the silages ranging from 4.5 to 12.0 g/kg DM, which is lower than the toxic level for ruminants (i.e., 20 g/kg DM) proposed by Rahman et al. (2013). Teutonico and Knorr (1985) found that amaranth varieties showed a large variation in total oxalate concentration (i.e., 2.0 to 114 g/kg DM) and this may be related to cultivar, plant maturity, climate, and field management (Bressani, 1993).

Tannin supplementation up to 40 g/kg DM in lambs’ diets showed no negative effects on ruminal fermentation or animal productivity in a long-term feeding trial (Salami et al., 2018). In the current study, the levels of TT in AMS were low, between 1.2 and 4.9 g/kg DM, and, therefore, had no adverse effect on the in vitro fermentation. Tannins may bind to protein in a ration and reduce its digestibility, but their effect is variable and may depend less on their concentration than their structure and molecular weight (Rochfort et al., 2008).

### Silage Fermentation Characteristics

Among the silages, the pH of CS was lower than that of the AMS because its higher WSC content led to higher lactic acid production (Kaiser et al., 2004). The AMS pH ranged from 4.0 to 4.6, which is considered low enough for an acceptable preservation (Faithfull, 2002). The pH of AMS from other cultivars was within the range of AMS obtained in the current study (Rezaei et al., 2014, 2015) and in the study of Seguin et al. (2013).

Protein degradation during fermentation is minimized in good quality silage (McDonald et al., 1991), and the lower level of NH_3_-N in CS (39.5 g/kg total N) compared with the (54 to 60 g/kg total N) in AMS probably resulted from its lower pH, as this reduces protein breakdown (Kaiser et al., 2004). The NH_3_-N concentration in AMS was relatively low for a low DM (<300 g/kg) silage (Demarquilly, 1990) indicating good preservation but Rezaei et al. (2009) reported lower NH_3_-N concentrations in low DM AMS.

Silage lactic acid levels (59 to 65 g/kg DM) in the present study were within the range of good quality silage (30 to 140 g/kg DM) characterized by ZoBell et al. (2004). In other work, higher lactic acid levels have been recorded for AMS (Seguin et al., 2013; Rezaei et al., 2014). Low levels of butyric acids (Table 4) in the AMS silages indicated that there had been good fermentation (Kaiser et al., 2004).

### In Vitro GP and Fermentation Variables

The in vitro ruminal pH values of the present treatments varied within the normal range of 5.9 to 7.0 reported by Dehority (2003), and the NH_3_-N levels were within the range for optimal growth of the rumen microbiota (>50 mg/L; Sinclair et al., 1993). The in vitro incubation of CS led to a lower NH_3_-N concentration compared with AMS, related to its lower CP level. Rumen VFA production depends on the levels of cellulose, hemicellulose, CP, and WSC in the feed (Bureenok et al., 2011). The in vitro ruminal total VFA levels differed among the treatments suggesting that the ruminal fermentability of OM differed among the silages. The greater degradation (TDS) of CS compared with AMS explains the higher total VFA concentrations from CS during in vitro fermentation (Van Soest, 1994).

The higher in vitro ruminal cellulolytic bacteria population of CS in comparison with AMS corresponds to its higher NDF substrate concentration (Van Soest, 1994).

The lower in vitro GP_24_, OMD, TDS, and ME in AMS compared with CS was possibly due to the lower NFC and cellulolytic bacteria numbers, and higher ADL concentration (McDonald et al., 2011). Amaranth varieties used in the current study had lower GP_24_, OMD, ME, and TDS than those observed by Sarmadi et al. (2016), and this may be due to their higher ash and ADL levels.

The PF_24_ values of AMS were between 2.75 and 4.41 mg/mL, within a theoretical range defined by Makkar (2010), but the value for CS was below this range. Feedstuffs that have a higher PF_24_ (mg degraded substrate divided by mg gas produced in vitro) have more degraded substrate incorporated into microbial mass (i.e., improved efficiency of MCP production; Makkar, 2010). Consequently, the MCP of AMS was higher than that of CS incubated in vitro. The lower in vitro methane production of AMS compared with CS relates to their lower NDFom concentration, as methane production positively correlates with levels of NDF (Pinares-Patino et al., 2007). The higher DUP in AMS compared with CS corresponded to the decline in in vitro methane, that is, lower rumen protein fermentation results in lower methane production (Preston et al., 2012; Ho Quang Do et al., 2013).

### In Situ Degradability of DM and CP

For DM degradability, the increased fraction B in AMS compared with CS could be related to their higher CP content (i.e., more CP for ruminal bacteria to ferment fraction B; McDonald et al., 2011). Van Soest (1994) observed that there was a correlation between ED and NFC; however, in the current study, such correlation was not obtained in the AMS silages. Compared with reports by Karimi-Rahjerdi et al. (2015) and Sarmadi et al. (2016), the AMS in this study had a lower ED of DM, which is probably due to its higher ADL content, as high ADL concentrations can limit ruminal degradability (Jung and Allen, 1995).

Data on ruminal CP degradability (i.e., protein quality) of AMS are limited. In this study, AMS contained less fraction A and more fraction B and DUP compared with CS and this may be due to more CP being bound to cell wall components (Abbasi et al., 2012). The higher DUP in AMS may be due to the presence of tannins reducing proteolytic bacteria activity in the in vitro fermentation (Molan et al., 2001).

Since yield was measured using one year and one location, further research is warranted.

## CONCLUSIONS

In comparison to CS, the ensiled amaranth from the cultivar A28 could be a potential forage resource for ruminants based on its high DM yield, CP and DUP content, with low antinutrient compounds, and in vitro methane production (i.e., A28 could be used instead of CS). However, more in vivo work is needed to assess the amaranth forage quality particularly in terms of intake.
